# Identification of risk factors for postoperative complications after right colectomy and low anterior resection in patients ≥85 years old with colorectal cancer using the National Clinical Database

**DOI:** 10.1002/ags3.12876

**Published:** 2024-11-11

**Authors:** Tomonori Akagi, Shiori Nishimura, Yoshitake Ueda, Masafumi Inomata, Hidefumi Shiroshita, Shuji Takiguchi, Yoshiharu Sakai, Hiraku Kumamaru, Hideki Ueno, Yuko Kitagawa

**Affiliations:** ^1^ Academic Committee of the Japan Society for Endoscopic Surgery Tokyo Japan; ^2^ Department of Gastroenterological and Pediatric Surgery, Faculty of Medicine Oita University Oita Japan; ^3^ Department of Healthcare Quality Assessment The University of Tokyo Graduate School of Medicine Tokyo Japan; ^4^ Department of Comprehensive Surgery for Community Medicine, Faculty of Medicine Oita University Oita Japan; ^5^ Department of Gastroenterological Surgery Graduate School of Medical Sciences, Nagoya City University Aichi Japan; ^6^ Japanese Red Cross Osaka Hospital Osaka Japan; ^7^ Database Committee The Japanese Society of Gastroenterological Surgery Tokyo Japan; ^8^ Department of Surgery National Defense Medical College Saitama Japan; ^9^ The Japanese Society of Gastroenterological Surgery Tokyo Japan; ^10^ Department of Surgery Keio University School of Medicine Tokyo Japan

**Keywords:** elderly patients, low anterior resection, National Clinical Database, postoperative complications, right hemicolectomy

## Abstract

**Aim:**

The purpose of this study was to evaluate factors associated with surgical outcomes and postoperative complications (Grade ≥3 by Clavien–Dindo classification) of right hemicolectomy (RH) and low anterior resection (LAR) for colorectal cancer in patients ≥85 years old.

**Methods:**

We retrospectively analyzed National Clinical Database (NCD) data on patients aged ≥85 years who underwent RH and LAR for colorectal cancer between 2017 and 2020. All possible preoperative factors were used to explore the risk factors for serious postoperative complication in these very elderly patients with colorectal cancer.

**Results:**

For RH, the operative mortality rate was 1.1% (98 cases), and the rate of serious postoperative complications was 5.2% (480 cases). Similarly, the mortality rate was 1.1% (27 cases), and the rate of serious complications (Clavien–Dindo Grade ≥3) was 8.7% (206 cases) for LAR. Based on multivariate analysis, independent risk factors for serious postoperative complications were male sex, ADL (partially dependent), hypertension, platelets (<150 000/μL), serum Na (<138 mEq/L), and PT‐INR (>1.1) for RH, and ASA‐PS (Grade ≥3), history of pneumonia, creatinine (>1.2 mg/day), and serum Na (<138 mEq/L) for LAR.

**Conclusions:**

The present results for RH and LAR suggest that surgical treatment for patients aged ≥85 years old is safe and feasible. Surgeons need to pay special attention more to physical status and past medical history than to tumor factors to prevent serious postoperative complications in these older patients with colorectal cancer.

## Graphical abstract

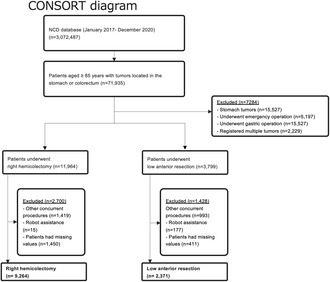

## 1 INTRODUCTION

With an average life expectancy of 81.09 years for men and 87.26 years for women in 2017, Japan is experiencing an unprecedented aging of its population.^1^ Globally, surgery and other treatments for elderly patients with colorectal cancer (CRC) are increasing.^2^ To optimize surgical outcomes, elderly patients need to be well informed prior to surgery.^3^ It is well known that preoperative frailty, comorbidities, decreased resilience and functional status, and lower Eastern Cooperative Oncology Group Performance Status (ECOG‐PS) are associated with an increased incidence of postoperative complications.^4^ Furthermore, once postoperative complications develop in the elderly, they are often fatal. However, few large‐scale studies have analyzed the relationship between preoperative general condition and outcomes in patients with CRC aged 85 years or older.^5^, ^6^ Flynn et al. compared the outcomes of CRC surgery between patients aged 85 years or older and those aged 75 years to less than 85 years old and reported no difference in short‐term outcomes between the two groups.^7^ Despite this finding, at least a decline in organ reserve is inevitable with aging.

To appropriately determine the choice of surgical technique for and assess the risk of surgical complications in patients aged 85 years or older, a large cohort study using real‐world data from Japan, which has entered the era of a hyper‐aged society, would be useful. Therefore, in this study based on real‐world data of patients aged 85 years or older who underwent surgery for CRC, we clarified clinicopathological factors and examined the association between preoperative factors including general condition and risk factors for postoperative complications.

## 2 METHODS

### 2.1 Data source and collection

All data were retrieved from the National Clinical Database (NCD) Gastroenterological section. The details of patient registration in this nationwide web‐based data entry system of the NCD were described previously.^8^, ^9^ As of 2018, over 5000 institutions have participated in the NCD system, with approximately 1 400 000 surgical cases, corresponding to more than 95% of all surgeries in Japan, being registered annually. Details input into the system for all surgical cases registered in the NCD include data on comorbidities, preoperative laboratory test results, postoperative complications, and mortality. The protocol for this study was approved by the institutional review board of Oita University (approval number 2119).

### 2.2 Study population

**FIGURE 1:**
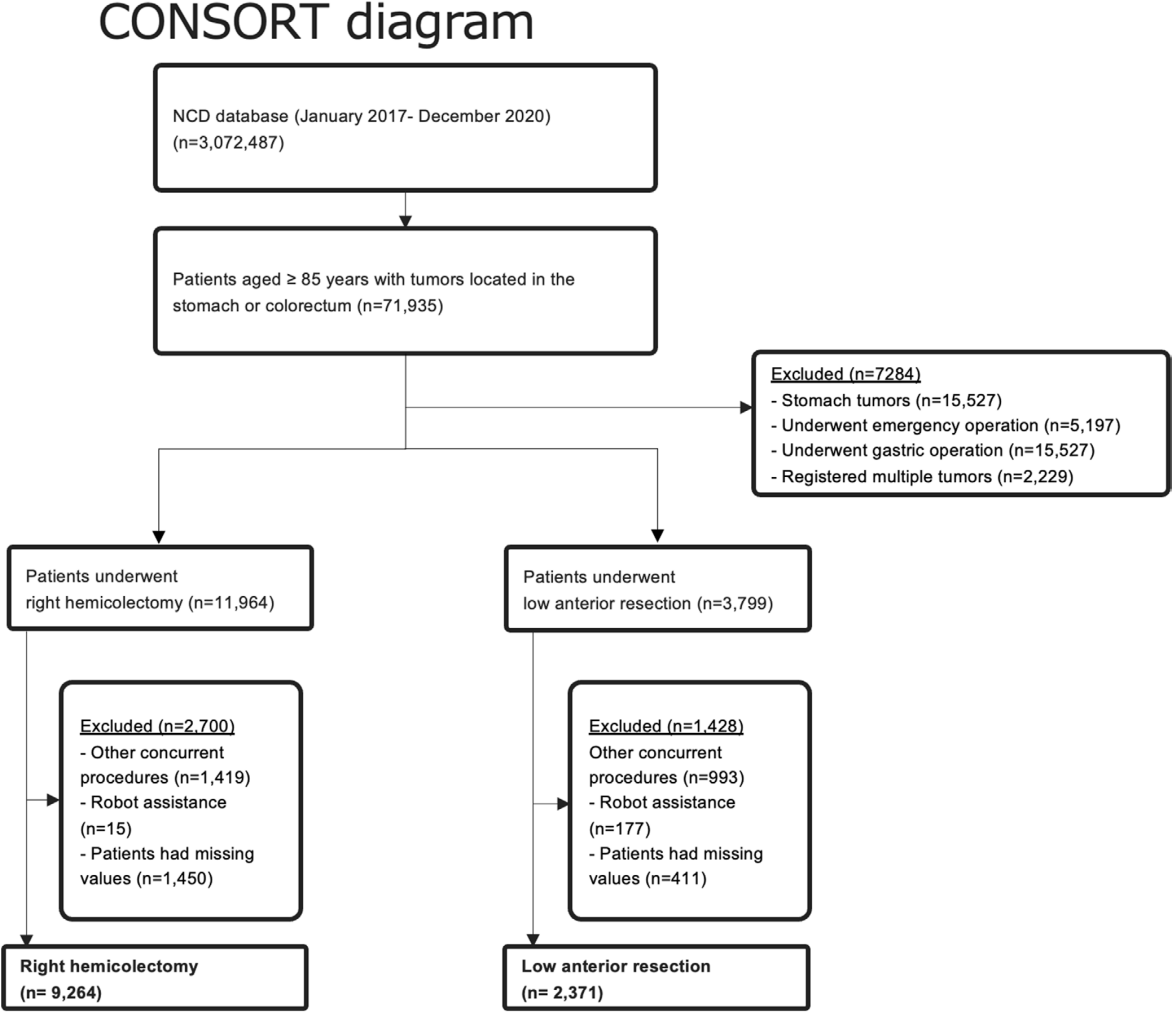
CONSORT diagram. Flowchart detailing the patient selection process for colorectal cancers.

Cases of right hemicolectomy (RH) and low anterior resection (LAR) registered in the NCD between January 2017 and December 2020 were included. Excluded from this study were patients with emergent surgery, stomach tumors, and multiple tumors registered, as well as those who underwent robotic‐assisted operation, other concurrent procedures including colostomy, and those with missing values (Figure 1).

### 2.3 Study endpoints

The primary endpoints of this study were the incidences of overall serious postoperative morbidity of Grade ≥3 according to the Clavien–Dindo classification,^10^ such as pancreatic fistula, postoperative pneumonia, and anastomotic leakage, which were evaluated as independent complications, and of overall complication and mortality (within 30 days after surgery).

### 2.4 Study analysis

We tabulated the background factors of the patients undergoing RH and LAR separately. We estimated the incidences of the outcomes in each procedure group as well as in the subgroups stratified by the background characteristics, and evaluated the strength of association of these risk factors with severe postoperative complications following colorectal surgery by using multivariable logistic regression analysis. The clinical factors included age at surgery (85–90, >90 years), sex (male vs. female), body mass index (BMI; <18.5, 18.5 to <25, ≥25 kg/m^2^), smoking history (Brinkman index: 0, 400 to <600, 600 to <1200, ≥1200), drinking habit (no, occasionally, habitual drinking), activities of daily living (independent, partially dependent, totally dependent), American Society of Anesthesiologists physical status (ASA‐PS; Grade ≤2 vs. ≥3), preoperative chemotherapy (yes vs. no), dyspnea (yes vs. no), diabetes mellitus (yes vs. no), chronic obstructive pulmonary disease (yes vs. no), pneumonia (yes vs. no), ascites (yes vs. no), hypertension (yes vs. no), ischemic heart disease (yes vs. no), previous percutaneous coronary intervention (yes vs. no), previous myocardial surgery (yes vs. no), previous surgery for peripheral vascular disease (PVD) (yes vs. no), symptom of PVD (yes vs. no), need for preoperative dialysis (yes vs. no), previous cerebrovascular disease (yes vs. no), disseminated cancer (yes vs. no), long‐term administration of steroid (yes vs. no), weight loss (yes vs. no), risk factor for bleeding (yes vs. no), preoperative blood transfusion (yes vs. no), previous surgical operation within 30 days, preoperative laboratory tests (WBC >9000/μL, hemoglobin [men: <13.5 g/dL; women: <11.5 g/dL], hematocrit [men: <37%; women: <32%], platelets <150 000/μL, serum albumin <4 g/dL, total bilirubin >1.2 mg/dL, AST >35 IU/L, ALT >35 IU/L, ALP >340 IU/L, BUN >12 mg/dL, creatinine >1.2 mg/dL, serum Na <138 mEq/L, APTT >40 s, PT‐INR >1.1), clinical T stage (T0/Tis, T1, T2, T3, T4, Tx), clinical N stage (N0, N1, N2, N3, Nx), and clinical M stage (M0, M1). The 7th edition of the American Joint Committee on Cancer TNM classification was used to extract representative tumor depth (T), node metastasis (N), distant metastasis (M), and surgical approach (laparoscopic; yes vs. no). All statistical analyses were performed with SAS version 9.4 (SAS Institute, Cary, NC, USA).

## 3 RESULTS

### 3.1 Patient characteristics

In total, 15 763 cases of colorectal surgery were identified from the NCD between January 2017 and December 2020. Among them, 9264 patients underwent RH and 2371 patients underwent LAR. Patient and tumor characteristics are shown in Table 1. RH was performed in 7372 (79.6%) patients aged 85–90 years old and in 1892 patients (20.4%) aged ≥90 years. Similarly, LAR was performed in 2040 (86.0%) patients aged 85–90 years old and in 331 patients (14.0%) aged ≥90 years. Laparoscopic RH was performed on 5680 patients (61.3%) and laparoscopic LAR was performed on 1752 patients (73.9%).

**TABLE 1:** Patient characteristics.

Characteristic	Right hemicolectomy	Low anterior resection
	*n* (%)	*n* (%)
Overall, *N*	9264	2371
Demographics		
Age, years		
85–90	7372 (79.6)	2040 (86.0)
>90	1892 (20.4)	331 (14.0)
Sex		
Men	3478 (37.5)	1127 (47.5)
Women	5786 (62.5)	1244 (52.5)
BMI, kg/m^2^		
Normal (18.5 to <25)	6117 (66.0)	1617 (68.2)
Underweight (<18.5)	1895 (20.5)	435 (18.3)
Overweight (≥25)	1252 (13.5)	319 (13.5)
Current smoker (within a year)	318 (3.4)	112 (4.7)
Drinking habit		
No	7180 (77.5)	1690 (71.3)
Occasionally	1100 (11.9)	359 (15.1)
Habitual drinking	984 (10.6)	322 (13.6)
Preoperative comorbidities and status		
ADL (within 30 days)		
Independent	7406 (79.9)	1988 (83.8)
Partially dependent	1535 (16.6)	336 (14.2)
Totally dependent	323 (3.5)	47 (2.0)
ASA‐PS		
Grade ≤2	6470 (69.8)	1764 (74.4)
Grade ≥3	2794 (30.2)	607 (25.6)
Preoperative chemotherapy	20 (0.2)	14 (0.6)
Dyspnea	268 (2.9)	63 (2.7)
COPD	335 (3.6)	115 (4.9)
Pneumonia	77 (0.8)	11 (0.5)
Ascites	286 (3.1)	19 (0.8)
Diabetes mellitus	1809 (19.5)	412 (17.4)
Hypertension	5414 (58.4)	1338 (56.4)
Heart failure (within 30 days)	263 (2.8)	51 (2.2)
Myocardial infarction (within 6 months)	60 (0.6)	15 (0.6)
Angina pectoris (within 30 days)	193 (2.1)	34 (1.4)
Previous PCI	359 (3.9)	85 (3.6)
Previous myocardiac surgery	159 (1.7)	38 (1.6)
Previous PVD surgery	54 (0.6)	19 (0.8)
Symptom of PVD	33 (0.4)	14 (0.6)
Dialysis (within 14 days)	32 (0.3)	8 (0.3)
History of cerebrovascular disease	763 (8.2)	161 (6.8)
Disseminated cancer	217 (2.3)	49 (2.1)
Long‐term administration of steroid	107 (1.2)	21 (0.9)
Body weight loss^a^	318 (3.4)	42 (1.8)
Risk factors for bleeding^b^	495 (5.3)	123 (5.2)
Preoperative blood transfusion (within 72 h)	564 (6.1)	69 (2.9)
Surgical operation (within 30 days)	50 (0.5)	20 (0.8)
Preoperative laboratory tests		
WBC >9000/μL	876 (9.5)	126 (5.3)
Hemoglobin, men: <13.5 g/dL; women: <11.5 g/dL	7112 (76.8)	1508 (63.6)
Hematocrit, men: <37%; women: <32%	5162 (55.7)	997 (42.0)
Platelets <150 000/μL	537 (5.8)	161 (6.8)
Serum albumin <4 g/dL	7064 (76.3)	1521 (64.2)
Total bilirubin >1.2 mg/dL	172 (1.9)	60 (2.5)
AST >35 IU/L	550 (5.9)	129 (5.4)
ALT >35 IU/L	280 (3.0)	81 (3.4)
ALP >340 IU/L	789 (8.5)	196 (8.3)
BUN >12 mg/dL	1974 (21.3)	537 (22.6)
Creatinine >1.2 mg/dL	1088 (11.7)	299 (12.6)
Serum Na <138 mEq/L	1703 (18.4)	271 (11.4)
APTT >40 s	401 (4.3)	97 (4.1)
PT‐INR >1.1	1108 (12.0)	229 (9.7)
Tumor factors		
T factor		
T0, Tis	109 (1.2)	20 (0.8)
T1	541 (5.8)	181 (7.6)
T2	844 (9.1)	394 (16.6)
T3	5062 (54.6)	1322 (55.8)
T4	2708 (29.2)	423 (17.8)
N factor		
N0	5358 (57.8)	1426 (60.1)
N1	2603 (28.1)	673 (28.4)
N2	1303 (14.1)	272 (11.5)
M factor		
M0	8531 (92.1)	2223 (93.8)
M1	733 (7.9)	148 (6.2)
Surgical approach		
Laparoscopy	5680 (61.3)	1752 (73.9)

Abbreviations: ADL, activities of daily living; ALP, alkaline phosphatase; ALT, alanine aminotransferase; APTT, activated partial thromboplastin time; ASA‐PS, American Society of Anesthesiologists physical status; AST, aspartate aminotransferase; BMI, body mass index; BUN, blood urea nitrogen; COPD, chronic obstructive pulmonary disease; PCI, percutaneous coronary intervention; PT‐INR, prothrombin time‐international normalized ratio; PVD, peripheral vascular disease; WBC, white blood cells.

^a^ Weight loss of 10% or more in the past 6 months.

^b^ Anticoagulant therapy.

### 3.2 Surgical outcomes in RH

Table 2 shows the postoperative events in both procedures. For RH, the operative mortality rate was 1.1% (98 cases). The incidence rate of overall postoperative complications was 28.4% (2628 cases), and the rate of serious postoperative complications was 5.2% (480 cases). Among all serious postoperative complications, anastomotic leakage, bowel obstruction, ileus, and postoperative pneumonia were observed in 81 (16.9%), 22 (4.6%), 67 (14.0%), and 72 (15.0%) patients, respectively.

**TABLE 2:** Postoperative events.

Postoperative event	Right hemicolectomy *n* (%)	Low anterior resection *n* (%)
Overall, *N*	9264	2371
Postoperative death within 30 days	98 (1.1)	27 (1.1)
Overall postoperative complications	2628 (28.4)	712 (32.6)
Postoperative severe complications	480 (5.2)	206 (8.7)

### 3.3 Surgical outcomes in LAR

Table 2 shows the postoperative events. For LAR, the operative mortality rate was 1.1% (27 cases). The incidence rate of overall postoperative complications was 32.6% (712 cases), and the rate of serious postoperative complications was 8.7% (206 cases). Among all serious postoperative complications, anastomotic leakage, bowel obstruction, ileus, and postoperative pneumonia were observed in 94 (45.6%), 11 (5.3%), 24 (11.7%), and 29 (14.8%) patients, respectively.

### 3.4 Risk factors for serious postoperative complications after RH

Table 3 shows the results of the multivariable analysis of risk factors associated with serious postoperative complications in RH. Male sex (odds ratio [OR] 1.64, 95% confidence interval [CI] 1.30 to 2.04), ADL (partially dependent) (OR 1.27, 95% CI 1.00 to 1.62), hypertension (OR 1.31, 95% CI 1.07 to 1.60), platelets (<150 000/μL) (OR 1.44, 95% CI 1.02 to 2.03), serum Na (<138 mEq/L) (OR 1.39, 95% CI 1.11 to 1.73), and PT‐INR (>1.1) (OR 1.36, 95% CI 1.06 to 1.76) were found to be strong risk factors for serious postoperative complications in RH.

**TABLE 3:** Multivariate analysis of risk factors.

Factor	Postoperative severe complication, event rate (%)	Adjusted odds ratio (95% confidence interval)	Postoperative severe complication, event rate (%)		Adjusted odds ratio (95% confidence interval)
Overall, *N*	480 (5.2)		206 (8.7)		
Demographics					
Age, years					
85–90	365 (5.0)	1 [Reference]	179 (8.8)		1 [Reference]
>90	115 (6.1)	1.08 (0.86–1.36)	27 (8.2)		0.88 (0.57–1.37)
Sex					
Women	248 (4.3)	1 [Reference]	85 (6.9)		1 [Reference]
Men	232 (6.7)	1.64 (1.30–2.04)	121 (10.7)		1.35 (0.94–1.92)
BMI, kg/m^2^					
Normal (18.5 to <25)	308 (5.0)	1 [Reference]	136 (8.4)		1 [Reference]
Underweight (<18.5)	110 (5.8)	1.07 (0.85–1.36)	42 (9.7)		1.15 (0.78–1.70)
Overweight (≥25)	62 (5.0)	1.06 (0.80–1.41)	28 (8.8)		1.17 (0.75–1.83)
Current smoker (within a year)	16 (5.0)	0.75 (0.44–1.27)	10 (8.9)		0.73 (0.36–1.47)
Drinking habit					
No	364 (5.1)	1 [Reference]	138 (8.2)		1 [Reference]
Occasionally	61 (5.5)	1.02 (0.76–1.37)	34	(9.5)	0.98 (0.64–1.51)
Habitual drinking	55 (5.6)	0.91 (0.66–1.25)	34 (10.6)		1.08 (0.69–1.68)
Preoperative comorbidities and status					
ADL (within 30 days)					
Independent	348 (4.7)	1 [Reference]	170 (8.6)		1 [Reference]
Partially dependent	106 (6.9)	1.27 (1.00–1.62)	31 (9.2)		0.92 (0.59–1.43)
Totally dependent	26 (8.0)	1.33 (0.86–2.08)	5 (10.6)		0.90 (0.33–2.44)
ASA‐PS					
Grade ≤2	288 (4.5)	1 [Reference]	129 (7.3)		1 [Reference]
Grade ≥3	192 (6.9)	1.20 (0.97–1.47)	77 (12.7)		1.48 (1.06–2.05)
Preoperative chemotherapy	1 (5.0)	0.70 (0.09–5.37)	3 (21.4)		2.16 (0.50–9.43)
Dyspnea	27 (10.1)	1.27 (0.81–1.99)	8 (12.7)		0.77 (0.31–1.90)
COPD	26 (7.8)	1.19 (0.76–1.85)	20 (17.4)		1.55 (0.86–2.81)
Pneumonia	11 (14.3)	1.84 (0.93–3.65)	4 (36.4)		5.20 (1.21–22.39)
Ascites	26 (9.1)	1.45 (0.94–2.23)	4 (21.1)		0.37 (0.05–2.92)
Diabetes mellitus	98 (5.4)	0.93 (0.73–1.18)	36 (8.7)		0.83 (0.56–1.24)
Hypertension	305 (5.6)	1.31 (1.07–1.60)	127 (9.5)		1.19 (0.87–1.64)
Heart failure (within 30 days)	26 (9.9)	1.30 (0.82–2.04)	5 (9.8)		0.75 (0.27–2.11)
Myocardial infarction (within 6 months)	4 (6.7)	1.04 (0.35–3.06)	1 (6.7)		0.54 (0.06–4.79)
Angina pectoris (within 30 days)	16 (8.3)	1.51 (0.87–2.62)	7 (20.6)		2.16 (0.84–5.57)
Previous PCI	19 (5.3)	0.71 (0.43–1.20)	11 (12.9)		0.89 (0.42–1.89)
Previous myocardiac surgery	11 (6.9)	1.02 (0.53–1.93)	3 (7.9)		0.51 (0.14–1.83)
Previous PVD surgery	4 (7.4)	1.25 (0.42–3.74)	6 (31.6)		3.21 (0.98–10.56)
Symptom of PVD	1 (3.0)	0.39 (0.05–3.12)	4 (28.6)		1.28 (0.30–5.56)
Dialysis (within 14 days)	3 (9.4)	1.04 (0.29–3.70)	1 (12.5)		0.91 (0.10–8.35)
History of cerebrovascular disease	43 (5.6)	0.83 (0.60–1.17)	20 (12.4)		1.29 (0.76–2.19)
Disseminated cancer	18 (8.3)	1.27 (0.72–2.25)	4 (8.2)		0.35 (0.10–1.29)
Long‐term administration of steroid	8 (7.5)	1.29 (0.62–2.71)	3 (14.3)		1.47 (0.39–5.57)
Body weight loss	22 (6.9)	1.02 (0.64–1.62)	4 (9.5)		0.84 (0.27–2.59)
Risk factors for bleeding	33 (6.7)	1.04 (0.70–1.54)	16 (13.0)		1.23 (0.67–2.27)
Preoperative blood transfusion (within 72 h)	47 (8.3)	1.35 (0.97–1.87)	6 (8.7)		0.88 (0.36–2.14)
Surgical operation (within 30 days)	4 (8.0)	1.14 (0.39–3.29)	2 (10.0)		1.52 (0.33–6.99)
Preoperative laboratory tests					
WBC >9000/μL	57 (6.5)	1.06 (0.79–1.43)	14 (11.1)		1.25 (0.68–2.30)
Hemoglobin, men: <13.5 g/dL; women: <11.5 g/dL	401 (5.6)	1.17 (0.86–1.60)	151 (10.0)		1.08 (0.70–1.67)
Hematocrit, men: <37%; women: <32%	308 (6.0)	0.98 (0.77–1.26)	110 (11.0)		1.11 (0.75–1.66)
Platelets <150 000/μL	43 (8.0)	1.44 (1.02–2.03)	20 (12.4)		1.36 (0.80–2.33)
Serum albumin <4 g/dL	400 (5.7)	1.19 (0.90–1.56)	148 (9.7)		1.15 (0.80–1.64)
Total bilirubin >1.2 mg/dL	16 (9.3)	1.61 (0.93–2.78)	2 (3.3)		0.23 (0.05–1.09)
AST >35 IU/L	37 (6.7)	0.94 (0.59–1.50)	15 (11.6)		1.21 (0.55–2.63)
ALT >35 IU/L	21 (7.5)	1.12 (0.62–2.05)	9 (11.1)		0.88 (0.33–2.33)
ALP >340 IU/L	53 (6.7)	1.13 (0.83–1.55)	14 (7.1)		0.63 (0.34–1.16)
BUN >12 mg/dL	120 (6.1)	0.97 (0.76–1.24)	49 (9.1)		0.75 (0.50–1.12)
Creatinine >1.2 mg/dL	82 (7.5)	1.25 (0.93–1.67)	39 (13.0)		1.57 (1.00–2.48)
Serum Na <138 mEq/L	132 (7.8)	1.39 (1.11–1.73)	38 (14.0)		1.60 (1.06–2.42)
APTT >40 s	33 (8.2)	1.16 (0.78–1.72)	11 (11.3)		0.85 (0.41–1.77)
PT‐INR >1.1	93 (8.4)	1.36 (1.06–1.76)	30 (13.1)		1.50 (0.95–2.38)
Tumor factors					
T factor					
T0, Tis	4 (3.7)	1.01 (0.33–3.06)	2 (10.0)		0.58 (0.12–2.71)
T1	18 (3.3)	1 [Reference]	13 (7.2)		1 [Reference]
T2	39 (4.6)	1.40 (0.79–2.50)	29 (7.4)		1.10 (0.55–2.22)
T3	254 (5.0)	1.44 (0.86–2.38)	109 (8.2)		1.18 (0.62–2.23)
T4	165 (6.1)	1.69 (0.99–2.88)	53 (12.5)		1.69 (0.84–3.42)
N factor					
N0	286 (5.3)	1 [Reference]	121 (8.5)		1 [Reference]
N1	126 (4.8)	0.79 (0.63–1.00)	60 (8.9)		0.91 (0.64–1.30)
N2	68 (5.2)	0.80 (0.59–1.08)	25 (9.2)		0.88 (0.53–1.46)
M factor					
M0	432 (5.1)	1 [Reference]	187 (8.4)		1 [Reference]
M1	48 (6.5)	1.08 (0.74–1.56)	19 (12.8)		1.73 (0.96–3.13)
Surgical approach					
Laparoscopy	267 (4.7)	0.90 (0.74–1.09)	153 (8.7)		1.06 (0.75–1.50)

Abbreviations: ADL, activities of daily living; ALP, alkaline phosphatase; ALT, alanine aminotransferase; APTT, activated partial thromboplastin time; ASA‐PS, American Society of Anesthesiologists physical status; AST, aspartate aminotransferase; BMI, body mass index; BUN, blood urea nitrogen; COPD, chronic obstructive pulmonary disease; PCI, percutaneous coronary intervention; PT‐INR, prothrombin time‐international normalized ratio; PVD, peripheral vascular disease; WBC, white blood cells.

### 3.5 Risk factors for serious postoperative complications after LAR

Table 3 also shows the results of the multivariable analysis of risk factors associated with serious postoperative complications in LAR. Here, ASA‐PS (Grade ≥3) (OR 1.48, 95% CI 1.06 to 2.05), history of pneumonia (OR 5.20, 95% CI 1.21 to 22.39), creatinine (>1.2 mg/dL) (OR 1.57, 95% CI 1.00 to 2.48), and serum Na (<138 mEq/L) (OR 1.60, 95% CI 1.06 to 2.42) were the strong risk factors for serious postoperative complications in LAR.

## 4 DISCUSSION

In this study, the rates of surgical mortality and serious postoperative complications in the CRC patients ≥85 years of age were 1.1% and 5.2% for RH and 1.1% and 8.7% for LAR, respectively. Furthermore, independent risk factors for serious postoperative complications were male sex, ADL (partially dependent), hypertension, platelets (<150 000/μL), serum Na (<138 mEq/L), and PT‐INR (>1.1) for RH, and ASA‐PS (Grade ≥3), history of pneumonia, creatinine (>1.2 mg/dL), and serum Na (<138 mEq/L) for LAR. Elderly patients often have age‐related dysfunction of various organs and comorbidities,^11^ and it is important to consider these factors when selecting surgical techniques and determining preoperative and postoperative management. Further, as postoperative complications in the elderly are often fatal, risk and prognostic factors for postoperative complications should also be clarified when considering surgical techniques and patient management. The safety and feasibility of surgical treatment for CRC patients over 85 years of age has not been adequately studied. To our knowledge, this is the largest multicenter study to date to determine the safety and feasibility of surgical treatment for these older CRC patients.

Although previous reports have found no significant difference in mortality between older and younger patients,^10^, ^12^ some investigators have reported higher mortality rates in older patients.^13^, ^14^, ^15^ Among these reports, Dekker and colleagues noted that although older CRC patients have high mortality, those who survive the first year have cancer‐related survival rates similar to those of younger patients; therefore, it is important that treatment of older CRC patients focus on perioperative care.^16^ Yoshida et al. and Hasegawa et al. reported from NCD data that mortality in RH cases of all ages in 2017 was 1.2% and 1.0% in LAR cases.^17^, ^18^ The present data show that the mortality for RH and LAR in the very elderly is equivalent to that when all ages are included. Our results for RH and LAR suggest that surgical treatment for very old patients is safe and feasible. However, the risk factors for severe complications identified in this study should be taken into consideration in the treatment of CRC. Additionally, unlike with CRC, Hartmann's operation or diverting stoma accompanied by anastomosis should be considered as a surgical option in rectal cancer when the patient has the risk factors identified in this study.

Over time, it has become widely accepted that patient frailty and physical fitness, rather than the age of the elderly patient, should be considered in surgical decision making.^19^, ^20^ Previous studies have described the association between frailty and short‐term outcomes but have not fully investigated the relationship with preoperative general condition.^21^ Fukuoka et al. compared surgical outcomes in 109 CRC surgery patients aged ≥85 years and in 1131 patients aged <85 years. Their study showed that overall morbidity was significantly higher in the older group (42.2% vs. 21.9%), as were the respective frequencies of pneumonia and thromboembolism (8.2% vs. 0.7% and 3.6% vs. 0.8%, respectively).^22^ In contrast, Cross et al. compared the surgical outcomes of 4600 CRC patients ≥80 years old with 15 863 patients <80 years old in Australia and New Zealand and reported no difference in surgical complications between the patients in these two countries.^15^ All of these previous reports were small, single‐center studies from overseas countries with different medical environments than Japan. Contrastingly, the present study is based on surgical results from more than 98% of surgical facilities in Japan, in addition to large, multicenter studies using big data, and is more in line with the real world in Japan. The risk factors for Grade 3 or higher complications in the present study are different from the CR‐POSSUM factors of cardiac function, blood pressure, hematolysis, hemoglobin, and urea.^23^, ^24^ However, the Comprehensive Geriatric Assessment (CGA), which is frequently used for preoperative screening, considers ADL and physical status, which were identified as risk factors in RH and LAR in the present study.^25^, ^26^ This indicates the importance of complementing perioperative risk assessment with the CGA, which evaluates elderly people from three aspects: “life function,” “mental and psychological,” and “social and environmental.” In addition, we found that some factors defining Grade 3 or higher serious complications were consistent with RH and LAR, whereas others were not. Sex and PT‐INR were the common risk factors for postoperative complications. Although the reasons for the discrepancies are not clear, it is important to at least keep these factors in mind during the perioperative management of patients undergoing either procedure. We believe that these factors will be important in the development of a calculation model for predicting postoperative complications in patients ≥85 years of age, and we plan to study them further in the future.

In the present study, laparoscopic surgery, a minimally invasive procedure, was not identified as a factor related to the reduction of Grade 3 or higher complications. Nakamura et al. reported the usefulness of laparoscopic colorectal resection compared to open surgery in patients aged ≥85 years,^27^ whereas in the present study patients, the laparoscopic approach was not an independent risk factor in either RH or LAR. However, we did not examine the typical parameters that are useful in laparoscopic surgery compared to open surgery, such as length of postoperative hospital stay and amount of blood loss. We consider that the laparoscopic approach is not contraindicated even for very old patients with CRC.

In CRC surgery cases in Japan, in addition to the increase in the number of elderly patients aged ≥75 years, the number of cases of patients aged ≥85 years is clearly increasing. In this context, the risk factors for complications extracted from real‐world data in Japan may be useful information for many colorectal surgeons when considering perioperative management and surgical procedure selection. In addition to the POSSUM‐score,^23^, ^24^ CGA, and Clinical Frailty Scale^28^ used for preoperative evaluation in daily practice, surgery based on the real‐world factors identified in this study in Japan is expected to improve outcomes.

This study has several limitations. First, patients in whom surgery was avoided cannot be evaluated by NCD items. It is possible that cases of patients at high risk for death or complications were avoided, thus resulting in more stable outcomes. Second is the lack of long‐term outcome evaluation because relevant data were not available in the NCD. Third, the impacts of perioperative management for preventing postoperative complications, such as pre‐rehabilitation, preoperative breathing training, and enhanced recovery after surgery, were not evaluated in this study. Prospective studies are needed in the near future to address these points.

In conclusion, our results of real‐world practice in Japan show that surgical treatment for patients aged ≥85 years with CRC is safe and feasible. We believe that age alone is not a contraindication to RH or LAR for CRC even in these very old patients. The risk factors for severe complications identified in this study should be taken into consideration when planning treatments. Surgeons and medical staff should pay special attention to these risk factors in older CRC patients when performing RH and LAR to avoid serious postoperative complications.

## AUTHOR CONTRIBUTIONS

Study concepts, design, and data interpretation: all authors. Data acquisition and analysis: Shiori Nishimura, Hiraku Kumamaru. Article preparation: all authors. Article review: Shuji Takiguchi, Yoshiharu Sakai, Hideki Ueno, Yuko Kitagawa.

## CONFLICT OF INTEREST STATEMENT

H.K. reports receiving consultation fees from EPS Corporation, and speaker fees from Chugai Pharmaceutical Co., Ltd. H.K. reports receiving research funding from Amgen K.K. for an unrelated research project. H.K. and S.N. are affiliated with the Department of Healthcare Quality Assessment at the University of Tokyo, a social collaboration department supported by the National Clinical Database, Johnson & Johnson K.K., Nipro Corporation, and Intuitive Surgical Sàrl. M.I. is an editorial board member of *Annals of Gastroenterological Surgery* dealing with the lower digestive tract. S.T. is an editorial board member of *Annals of Gastroenterological Surgery* dealing with the general. H.U. is an associate editor of the *Annals of Gastroenterological Surgery* dealing with the lower digestive tract. Y.K. is Editor‐in‐Chief of *Annals of Gastroenterological Surgery*. The remaining authors declare no conflicts of interest for this article.

## ETHICS STATEMENT

Approval of research protocol: The study protocol was approved by the institutional review board of Oita University (approval number: 2119).

Informed consent: N/A.

Registry and the Registration No. of the study/trial: N/A.

Animal Studies: N/A.
